# Chondroitin sulfates play a major role in breast cancer metastasis: a role for *CSPG4 *and *CHST11 *gene expression in forming surface P-selectin ligands in aggressive breast cancer cells

**DOI:** 10.1186/bcr2895

**Published:** 2011-06-09

**Authors:** Craig A Cooney, Fariba Jousheghany, Aiwei Yao-Borengasser, Bounleut Phanavanh, Tina Gomes, Ann Marie Kieber-Emmons, Eric R Siegel, Larry J Suva, Soldano Ferrone, Thomas Kieber-Emmons, Behjatolah Monzavi-Karbassi

**Affiliations:** 1 Department of Biochemistry and Molecular Biology, the University of Arkansas for Medical Sciences, 4301 W. Markham St., Little Rock, Arkansas 72205, USA; 2 Central Arkansas Veterans Healthcare Systems, 4300 West 7th St., Little Rock, Arkansas 72205, USA; 3 Winthrop P. Rockefeller Cancer Institute, University of Arkansas for Medical Sciences, 4301 W. Markham St., Little Rock, AR 72205, USA; 4 Department of Pathology, University of Arkansas for Medical Sciences, 4301 W. Markham St., Little Rock, AR 72205, USA; 5 Department of Biostatistics, University of Arkansas for Medical Sciences, 4301 W. Markham St., Little Rock, AR 72205, USA; 6 Department of Orthopaedic Surgery and Center for Orthopaedic Research, University of Arkansas for Medical Sciences, 4301 W. Markham St., Little Rock, AR 72205, USA; 7 University of Pittsburgh Cancer Institute, Departments of Surgery, of Immunology, of Pathology, Cancer Immunology Program, School of Medicine, University of Pittsburgh, 5117 Centre Ave, Pittsburgh, PA 15232, USA

## Abstract

**Introduction:**

We have previously demonstrated that chondroitin sulfate glycosaminoglycans (CS-GAGs) on breast cancer cells function as P-selectin ligands. This study was performed to identify the carrier proteoglycan (PG) and the sulfotransferase gene involved in synthesis of the surface P-selectin-reactive CS-GAGs in human breast cancer cells with high metastatic capacity, as well as to determine a direct role for CS-GAGs in metastatic spread.

**Methods:**

Quantitative real-time PCR (qRT-PCR) and flow cytometry assays were used to detect the expression of genes involved in the sulfation and presentation of chondroitin in several human breast cancer cell lines. Transient transfection of the human breast cancer cell line MDA-MB-231 with the siRNAs for carbohydrate (chondroitin 4) sulfotransferase-11 (*CHST11*) and chondroitin sulfate proteoglycan 4 (*CSPG4 *) was used to investigate the involvement of these genes in expression of surface P-selectin ligands. The expression of *CSPG4 *and *CHST11 *in 15 primary invasive breast cancer clinical specimens was assessed by qRT-PCR. The role of CS-GAGs in metastasis was tested using the 4T1 murine mammary cell line (10 mice per group).

**Results:**

The *CHST11 *gene was highly expressed in aggressive breast cancer cells but significantly less so in less aggressive breast cancer cell lines. A positive correlation was observed between the expression levels *of CHST11 *and P-selectin binding to cells (*P *< 0.0001). Blocking the expression of *CHST11 *with siRNA inhibited CS-A expression and P-selectin binding to MDA-MB-231 cells. The carrier proteoglycan CSPG4 was highly expressed on the aggressive breast cancer cell lines and contributed to the P-selectin binding and CS-A expression. In addition, *CSPG4 *and *CHST11 *were over-expressed in tumor-containing clinical tissue specimens compared with normal tissues. Enzymatic removal of tumor-cell surface CS-GAGs significantly inhibited lung colonization of the 4T1 murine mammary cell line (*P *= 0.0002).

**Conclusions:**

Cell surface P-selectin binding depends on *CHST11 *gene expression. CSPG4 serves as a P-selectin ligand through its CS chain and participates in P-selectin binding to the highly metastatic breast cancer cells. Removal of CS-GAGs greatly reduces metastatic lung colonization by 4T1 cells. The data strongly indicate that CS-GAGs and their biosynthetic pathways are promising targets for the development of anti-metastatic therapies.

## Introduction

Tumor-associated glycans play a significant role in promoting aggressive and metastatic behavior of malignant cells [1-5], participating in cell-cell and cell-extracellular matrix interactions that promote tumor cell adhesion and migration. Among glycans that play a critical role in stromal tumor cell interactions are glycosaminoglycans (GAGs) attached to proteoglycans (PGs). Altered production levels of PGs and structural changes in their GAGs are reported in many neoplastic tissues [6-10]. GAGs are polysaccharide chains covalently attached to protein cores that together comprise PGs [6,11] and based on the prevalence of GAG chains, chondroitin sulfate (CS)/dermatan sulfate (DS) PGs (CS/DS-PGs), heparan sulfate PGs and keratan sulfate PGs have been described [12]. Increased production of CS/DS-GAGs is found in transformed fibroblasts and mammary carcinoma cells [8,13,14] and it has been shown that these polysaccharides contribute to fibrosarcoma cell proliferation, adhesion and migration [15].

Several studies have disclosed the critical involvement of P-selectin in the facilitation of blood borne metastases [16-18]. P-selectin/ligand interaction often requires sialylated and fucosylated carbohydrate such as sialyl Lewis X and sialyl Lewis A [19]; however, P-selectin also binds to heparan sulfate, certain sulfated glycolipids and CS/DS-GAGs [20-23]. In previous studies we found that CS/DS-GAGs are expressed on the cell surface of murine and human breast cancer cell lines with high metastatic capacity and that they play a major role in P-selectin binding and P-selectin-mediated adhesion of cancer cells to platelets and endothelial cells [24]. However, variation in the abundance and function of CS/DS relative to tumor cell phenotypic properties and P-selectin binding are not well defined. It is likely that P-selectin binding to tumor cells and the functional consequences of such binding are dependent on which sulfotransferases define the relevant CS/DS and which core proteins carry the CS polysaccharide.

CS/DS expression is controlled by many enzymes in a complex biosynthetic pathway and this leads to considerable variation in structure and function. The chondroitin backbone of CS/DS-GAGs consists of repetitive disaccharide units containing D-glucuronic acid (GlcA) and N-acetyl-D-galactosamine (GalNAc) residues, or varying proportions of L-iduronic acid (IdoA) in place of GlcA [25,26]. Major structural variability of the CS/DS chains is due to the sulfation positions in repeating disaccharide units by the site-specific activities of sulfotransferases that produce the variants CS-A, CS-B (dermatan sulfate, DS), CS-C, CS-D and CS-E [26,27]. CHST3, CHST7, CHST11, CHST12, CHST13, CHST14 and CHST15 are the enzymes that sulfate the GalNAc residues of chondroitin polymers. The expression levels of these enzymes are hypothesized to affect the production of P-selectin ligands. CHST11 and CHST13 share specificity, as they mediate 4-O sulfation of chondroitin [28-30]. CHST12 and CHST14 mediate mostly 4-O sulfation of DS units [30,31]. CHST3 and CHST7 share specificity for 6-O sulfation of chondroitin [32,33]. CHST15 transfers sulfate to the carbon-6 of an already 4-O sulfated GalNAc residue producing oversulfated CS-E [34]. The relationship between the relative expression of these sulfotransferases with the expression of P-selectin-reactive CS/DS-GAGs has not been reported and is addressed in this paper.

The variation, abundance and function of CS/DS-GAGs are also affected by the expression of the PG core protein presenting them. Syndecan-1 (SDC-1), syndecan-4 (SDC-4), neuropilin-1 (NRP-1) and CSPG4 are considered major pro-malignancy membrane proteins capable of carrying CS chains [35-39]. Among these PGs, CSPG4 exclusively carries CS chains [40,41]. CSPG4 is a human homolog of Rat NG2, which is also known as High Molecular Weight Melanoma Associated Antigen and Melanoma Chondroitin Sulfate Proteoglycan [40,42,43]. This tumor-associated cell surface PG potentiates cell motility and promotes invasiveness and the metastatic potential of tumor cells in melanoma [44-46]. CSPG4 is also linked to cancer stem cells via signaling mechanisms [39,45]. Therefore, studying whether this PG interacts directly with P-selectin is of particular interest.

The expression of CSPG4 in breast cancer has been reported very recently [39]. Depending on its relative expression levels, this PG might be a major core protein presenting CS-GAGs on an aggressive subset of tumor cells, interacting with P-selectin. In the current study we investigated whether CSPG4, via its CS/DS chain can serve as a P-selectin ligand and whether expression of specific chondroitin sulfotransferases contributes to P-selectin interaction with cells. We further examined the involvement of CS-GAG chains in lung colonization by the 4T1 mammary cell line with and without enzymatic removal of CS-GAGs in a murine experimental metastasis model. Our data show for the first time that P-selectin can bind to tumor cells via CSPG4 and that *CHST11 *expression is linked to P-selectin-reactive cell surface CS/DS-GAGs. The results directly link CS/DS-GAGs to the metastatic spread of breast cancer. These findings have significant implications for understanding mechanisms of breast cancer metastasis, paving the way towards developing alternative strategies to treat and prevent metastasis.

## Materials and methods

### Reagents

Anti-CS-A mAb 2H6 was from Associates of Cape Cod/Seikagaku America (Falmouth, MA, USA), anti-CSPG4 mAb 225.28 was made as described [47,48], and recombinant human P-selectin/Fc (human IgG) was from R&D Systems (Minneapolis, MN, USA). Fluorescence-conjugated anti-human IgG, anti-mouse IgM and chondroitinase ABC were from Sigma (St. Louis, MO, USA). R-Phycoerythrin-conjugated polyclonal goat anti-mouse F(ab')_2 _fragment was from Dako North America, Inc. (Carpinteria, CA, USA). DNA primers were from Integrated DNA Technologies (IDT, Coralville, IA, USA). Real-time PCR reagents were from Applied Biosystems (Foster City, CA, USA). Pre-designed siRNA sequences were from Ambion (Austin, TX, USA) and Santa Cruz Biotechnology Inc. (Santa Cruz, CA, USA). siPORT™ *NeoFX*™ Transfection Agent was from Ambion. TRIzol reagent was from Invitrogen (Carlsbad, CA, USA).

### Cell lines and tissue specimens

Human breast cancer MCF7, MDA-MB-231, MDA-MB-468 cell lines and the murine 4T1 cell line were from ATCC (Manassas, VA, USA). Human MDA-MET cells were selected *in vivo *for their bone colonizing phenotype [49]. 4T1 cells were used within 10 passages and less than six months after receipt. We confirmed cell line identities by the Human Cell Line Authentication test (Genetica DNA Laboratories, Inc. Cincinnati, OH, USA). The melanoma cell lines M14 and M14-CSPG4, stably transfected to express CSPG4, were used as homologous CSPG4-non-expressing and expressing cell lines [50] and were characterized by real-time PCR for CSPG4 expression. Cells were cultured in a base medium supplemented with 10% heat-inactivated fetal bovine serum (Life Technologies, Carlsbad, CA, USA), 50 units/mL penicillin, and 50 μg/mL streptomycin. Base medium for MDA-MB-231, MDA-MET, MDA-MB-468, and 4T1 was DMEM (Fisher Scientific, Pittsburgh, PA, USA). For MCF7 base medium was MEM (Fisher Scientific) supplemented with 0.1 mM non-essential amino acids, 1 mM sodium pyruvate, and 0.01 mg/ml insulin (Invitrogen). For M14 and M14/CSPG4 RPMI 1640 medium (Fisher Scientific) was used with the addition of 500 μg/ml G418 (Invitrogen). Cells are checked every six months to be free from *Mycoplasma *contamination using the MycoAlert^® ^Mycoplasma Detection Kit (Lonza Rockland Inc., Rockland, ME, USA).

De-identified frozen specimens from 15 female breast cancer patients diagnosed with invasive ductal carcinoma were provided by the University of Arkansas for Medical Sciences (UAMS) Tissue Procurement Facility. Specimens were matched for each donor to have both tumor-free and tumor-containing breast tissues. According to the policy of the Tissue Procurement Facility, patients signed consent forms to donate excess specimen tissue and to allow use of related pathological data for research purposes, which were approved by the UAMS Institutional Review Board. For this study, an active human tissue use protocol approved by the UAMS Institutional Review Board was used. In this protocol, informed consent was waived due to the use of de-identified specimens with no link to patient identifiers.

### Total RNA isolation and qRT-PCR

Total RNA was isolated from cultured cells and tumor tissues using TRIzol reagent, following the manufacturer's instructions. The quantity and quality of the isolated RNA was determined by Agilent 2100 Bioanalyzer (Palo Alto, CA, USA). One μg of total RNA was reverse-transcribed using random-hexamer primers with TaqMan Reverse Transcription Reagents (Applied Biosystems). Reverse-transcribed RNA was amplified with SYBR Green PCR Master Mix (Applied Biosystems) plus 0.3 μM of gene-specific upstream and downstream primers during 40 cycles on an Applied Biosystems 7500 Fast Realtime cycler. Data were analyzed by absolute and relative quantification. In absolute quantification, data were expressed in relation to 18S RNA, where the standard curves were generated using pooled RNA from the samples assayed. In relative quantification, the 2 (^-Delta Delta C(T)^) method was used to assess the target transcript in a treatment group relative to that of an untreated control group using expression of an internal control (reference gene) to normalize data [51]. Expression of *GAPDH *and *β-actin *was used as internal control. Each cycle consisted of denaturation at 95°C for 15 s, and annealing and extension at 60°C for 60 s. The primer sequences are shown in Table 1.

**Table 1:** Primer and siRNA sequences used in this study

Primer	Sequence
18S forward 18S reverse	5' TTCGAACGTCTGCCCTATCAA 3' 5' ATGGTAGGCACGGCGACTA 3'
β-actin forward β-actin reverse	5' GACTTCGAGCAAGAGATGGCCAC3' 5' CAATGCCAGGGTACATGGTGGTG3'
CHST3 forward CHST3 reverse	5' GACTTTGTGCACAGCCTGAA 3' 5' AGAGCTTGGGGAATCTGCTT 3'
CHST7 forward CHST7 reverse	5' GGGGCAATCTGTCACACTCT 3' 5' AATTGCACAGCAGTTGTTGG 3'
CHST11 forward CHST11 reverse	5' TCCCTTTGGTGTGGACATCT 3' 5' CACGTGTCTGTCACCTGGTC 3'
CHST12 forward CHST12 reverse	5' GCACACGTCCTTCTCTAGGC 3' 5' AAACTCGTCGACATCGGAGT 3'
CHST13 forward CHST13 reverse	5' CCGGCATTTGGAAACAGAG 3' 5' GGGTCCTGATCCAGGTCATA 3'
CHST14 forward CHST14 reverse	5' TCTGATCCCCCATTTATCCA 3' 5' GGAGCAGAAAGGCACAAAAG3'
CHST15 forward CHST15 reverse	5' TGAGTTCACGACCAGACAGC 3' 5' CGTACCAACAGGGGCTCTTA 3'
CSPG4 forward CSPG4 reverse	5' CACACAGAGGAACCCTCGAT 3' 5' CTTCAGCGAGAGGAGCACTT 3'
ER1 forward ER1 reverse	5' AGGTGGGATACGAAAAGACCG 3' 5' AAGGTTGGCAGCTCTCATGTC 3'
GAPDH forward GAPDH reverse	5' ACAGTCAGCCGCATCTTCTT 3' 5' ACGACCAAATCCGTTGACTC 3'
NRP-1 forward NRP-1 reverse	5' CAAGGTGTTCATGAGGAAGTTCAA 3' 5' CCGCAGCTCAGGTGTATCATAGT 3'
SDC-1 forward SDC-1 reverse	5' GCTCTGGGGATGACTCTGAC 3' 5' GGTCTGCTGTGACAAGGTGA3'
SDC-4 forward SDC-4 reverse	5' GTCTGGCTCTGGAGATCTGG3' 5' CACCAAGGGATGGACAACTT3'
**siRNA**	**Sequence**
#31 #32 #33	UCAGUAGUUCUCGUAGACUtt UUCUGGGUGAACUUGUUGCgg AUCGAGUUUGUAGACUUCGta

18S, 18S ribosomal RNA; CHST3, Carbohydrate (chondroitin 6) sulfotransferase 3; CHST7, Carbohydrate (N-acetylglucosamine 6-O) sulfotransferase 7; CHST11, Carbohydrate (Chondroitin 4) Sulfotransferase 11; CHST12, Carbohydrate (Chondroitin 4) Sulfotransferase 12; CHST13, Carbohydrate (Chondroitin 4) Sulfotransferase 13; CHST14, Carbohydrate (N-acetylgalactosamine 4-O) Sulfotransferase 14; CHST15, Carbohydrate (N-acetylgalactosamine 4-sulfate 6-O) Sulfotransferase 15; CSPG4, Chondroitin Sulfate Proteoglycan 4; ER1, Estrogen Receptor 1; GAPDH, Glyceraldehyde-3-phosphate dehydrogenase; NPR-1, Neuropilin-1; SDC-1, Syndecan-1; SDC-4, Syndecan-4; siRNA, short interfering RNA.

### Flow cytometry

Binding of the recombinant human P-selectin/Fc molecule, anti-CS-A (2H6) and anti-CSPG4 (225.28) mAb to cells was determined using flow cytometry as previously described [24]. Briefly, cells were incubated with mAb or P-selectin/Fc, washed and stained with FITC-conjugated anti-mouse IgM or anti-human IgG prior to binding detection by flow cytometry. R-phycoerythrin-conjugated polyclonal goat anti-mouse F(ab')_2 _was used as secondary for detection of anti-CSPG4 binding.

### Switching off gene expression with siRNA

Three pre-designed siRNA sequences for *CHST11 *(Ambion) were used (Table 1). NG2 siRNA (sc-40771) from Santa Cruz Biotechnology, Inc. was used for inhibition of CSPG4. These sequences were transfected into cells growing in tissue culture using the siPORT™ *NeoFX*™ Transfection Agent, and mRNA levels were determined 48 hours later by real-time PCR. Expression of *GAPDH *was used as reference control. Reactivity of anti-CS-A mAb 2H6 and human recombinant P-selectin was assessed 144 hours after siRNA transfection. Transfection with *GAPDH *siRNA (Ambion) was used as control.

### Mice and tumor models

BALB/c female mice (six to eight weeks old) were from Harlan Laboratories (Indianapolis, IN, USA). We used 4T1 cells in an experimental metastasis model [24]. Cells were treated with chondroitinase ABC in HBSS buffer with protease inhibitors [24], or with the buffer and protease inhibitor alone (no chondroitinase ABC treatment) before inoculation through the tail vein. Each mouse (10 mice per group) received 2 × 10^4 ^4T1 cells. Mice were sacrificed 25 days after tumor cell injection and lungs were harvested to determine clonogenic cells by growing cells in medium containing 6-thioguanine [52]. Animal studies have been reviewed and approved by the Institutional Care and Use Committee of UAMS.

### Statistical analysis

For comparison of gene expression between cell lines, the raw amount for each mRNA was normalized to the control mRNA (18S) amount and then log transformed, and analyzed via one-way ANOVA with Tukey's *post-hoc *procedure. For the tissue comparisons of gene expression in patient samples, the tumor/normal expression ratios for each patient were log-transformed and subjected to one-sample t-tests. For siRNA effects on relative mRNA levels mean fluorescence intensities of antibody binding were log transformed and analyzed via ANOVA and Tukey's *post-hoc *procedure. Associations between mean fluorescence intensities of P-selectin binding and quantities of sulfotransferase transcripts were characterized using Pearson correlation tests on log-transformed data. In order to check the validity of assumptions for running Pearson's test, Spearman correlation analysis was also performed and when correlation coefficients were similar, assumptions were considered valid. For clonogenic assay comparisons, the number of clonogenic lung metastases was analyzed between groups by the Wilcoxon rank-sum test.

## Results

### *CHST11 *is overexpressed in aggressive human breast cancer cell lines and its expression correlates with P-selectin binding

We have shown that CS/DS-GAGs expressed on the cell surface of MDA-MB-231 and MDA-MET human breast cancer cells function as P-selectin ligand and that exogenous CS-E efficiently inhibits P-selectin binding to cells [24]. Among the major sulfotransferases able to sulfate the GlaNAC residue of the chondroitin disaccharide, CHST11, CHST12, and CHST13 are chondroitin 4-sulfotransferases, able to catalyze sulfation of chondroitin on the carbon-4 position of GalNAc sugars in disaccharide GAG units, producing primarily CS-A units (GlcAβ1-3GalNAc (4-SO_4_)) [28-30]. CHST14, dermatan 4-sulfotransferase 1 (D4ST-1), is specific for 4-O sulfation of the GalNAc in CS-B (DS) units after dermatan sulfate epimerase activity and its specificity can be shared by CHST12 [30,31]. CHST3 and CHST7 are chondroitin 6-sulfotransferases that transfer sulfate to carbon-6 position of GalNAc, competing with 4-sulfotransferases for the same substrate, producing primarily CS-C units (GlcAβ1-3GalNAc (6-SO_4_)) [32,33]. CHST15, GalNAc 4-sulfate 6-O-sulfotransferase (GalNAc4S-6ST), transfers sulfate to the carbon-6 of a 4-sulfated GalNAc, producing CS-E (GlcAβ1-3GalNAc (4-, 6-SO_4_)) [34].

**Figure 1:**
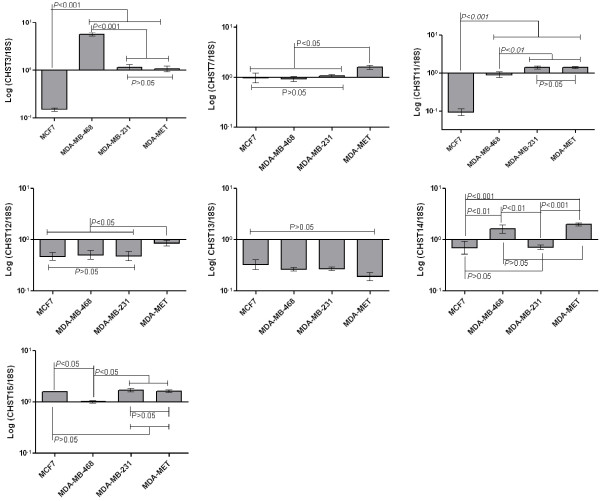
**The expression of the chondroitin sulfotransferase genes, in the breast cancer cells used**. The expression of *CHST3*, *CHST7*, *CHST11, CHST12, CHST13, CHST14, CHST15 *was measured by qRT-PCR and normalized using18S. Data were analyzed by one-way ANOVA and *post hoc *analysis using data from three (CHST7, CHST12, CHST13, CHST14) and four (CHST3, CHST11, CHST15) independent experiments. Means and standard deviations are shown. Statistically significant differences and *P *values are shown.

Quantitative real-time PCR was used to monitor the expression levels of these genes in several human breast cancer cell lines differing in their cancer phenotype. The human breast cancer cell lines MCF7, MDA-MB-468, MDA-MB-231, and MDA-MET represent increasing aggressiveness and metastatic capacity (MCF7 < MDA-MB-468 < MDA-MB-231 = MDA-MET). While expression of all genes was detected in these breast cancer cells, *CHST13 *expression was observed to be very low with no significant differences in expression between cell lines (Figure 1). Among the seven sulfotransferases tested, only *CHST11 *expression levels trended well with the metastatic potential of cells, ranging from a low in MCF7, intermediate to high in MDA-MB-468 and high in MDA-MB-231 and MDA-MET cells (Figure 1). The expression level of *CHST11 *was elevated with the increase in metastatic potential of cells. Such a trend was not observed with the expression levels of other genes examined. Therefore, the data may implicate the immediate product of *CHST11 *expression, CS-A, in the metastatic behavior of these cells.

**Figure 2:**
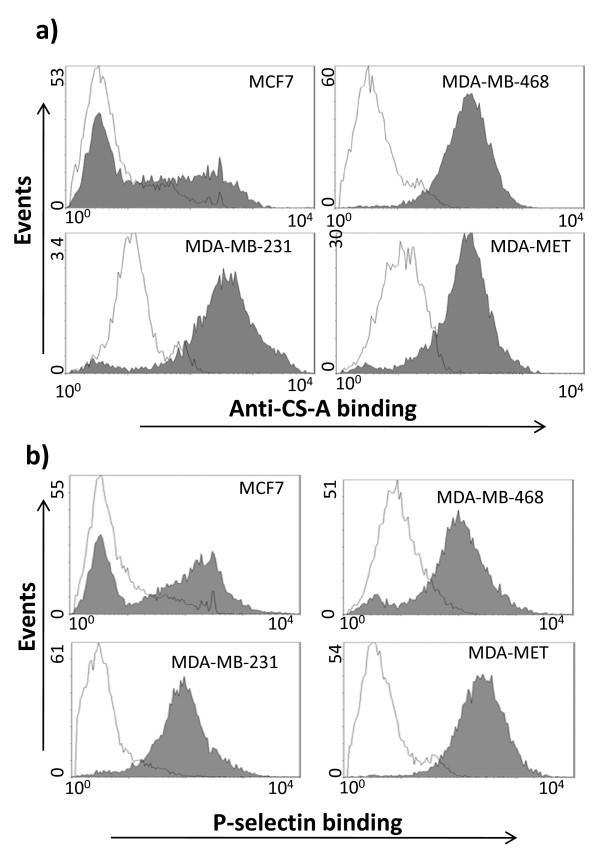
**Anti-CS-A mAb and P-selectin showed comparable binding to the cell lines**. **A) **Flow cytometry analysis of CS-A expression using anti-CS-A 2H6 mAb (15 μg/ml). **B) **P-selectin binding to cells using recombinant P-selectin (15 μg/ml). Open histograms show binding of the secondary antibody only (control), while filled histograms show anti-CS-A and P-selectin binding. One representative experiment out of three is shown.

Flow cytometry analysis of the binding of the anti-CS-A mAb 2H6, consistent with qRT-PCR data, indicates that the expression of CS-A was lower in the least aggressive MCF7 cell line and high in the most aggressive MDA-MB-231 and MDA-MET cell lines (Figure 2A). While MDA-MB-468 showed higher expression of CS-A than MCF7 cells, the CS-A expression in MDA-MB-468 was not different than its expression in MDA-MB-231 and MDA-MET. The lack of a difference in CS-A between these three cell lines could be due to the expression level of *CHST15 *that can turn the 4-sulfated product of *CHST11 to *CS-E. The expression of *CHST15 *was significantly lower in MDA-MB-468 than in MDA-MB-231 or MDA-MET (Figure 1) making the conversion of some CS-A to CS-E much more likely in MDA-MB-231 and MDA-MET than in MDA-MB-468. Among the sulfotransferase genes examined, P-selectin binding to these cells correlated the best with *CHST11 *expression. We performed a correlation analysis of the gene expression levels and the mean fluorescence intensities of P-selectin binding among all cell lines studied and observed that only *CHST11 *(r = 0.85, *P *< 0.0001) showed a statistically significant correlation with P-selectin binding (Table 2). P-selectin reactivity with cell lines was comparable to anti-CSA 2H6 binding (Figure 2A, B). These data suggest that *CHST11 *(a chondroitin-4-sulfotransferase) expression in these cells is associated with the production of P-selectin ligands.

**Table 2:** Correlation coefficients between P-selectin binding to cells and the expression of the sulfotransferases studied

Sulfotransferase gene	Pearson r	number	*P *value (two-tailed)
*CHST3*	0.25	16	0.36
*CHST7*	0.45	12	0.14
***CHST11***	**0.85**	**16**	**<0.0001**
*CHST12*	0.32	12	0.30
*CHST13*	-0.37	12	0.24
*CHST14*	0.20	12	0.52
*CHST15*	0.29	16	0.31

Gene expression was measured by qRT-PCR and normalized using 18S transcript. Mean Fluorescence Intensities of P-selectin binding to cells was assayed by flow cytometry. Cell lines were MCF7, MDA-MB-468, MDA-MB-231, and MDA-MET. Each assay was repeated independently 3 times per cell line for *CHST7, CHST12, CHST13 *and *CHST14 *and 4 times per cell line for *CHST3, CHST11, CHST15*. Then the data were log transformed and subjected to correlation analysis using both Pearson (parametric) and Spearman (nonparametric) correlation tests in order to check validity of assumptions. Correlation coefficients were similar, assumptions were considered valid, and the Pearson coefficient was reported. CHST3, Carbohydrate (chondroitin 6) sulfotransferase 3; CHST7, Carbohydrate (N-acetylglucosamine 6-O) sulfotransferase 7; CHST11, Carbohydrate (Chondroitin 4) Sulfotransferase 11; CHST12, Carbohydrate (Chondroitin 4) Sulfotransferase 12; CHST13; Carbohydrate (Chondroitin 4) Sulfotransferase 13; CHST14, Carbohydrate (N-acetylgalactosamine 4-O) Sulfotransferase 14; CHST15, Carbohydrate (N-acetylgalactosamine 4-sulfate 6-O) Sulfotransferase 15.

### Inhibition of *CHST11 *expression results in inhibition of CS-A production and P-selectin binding

**Figure 3:**
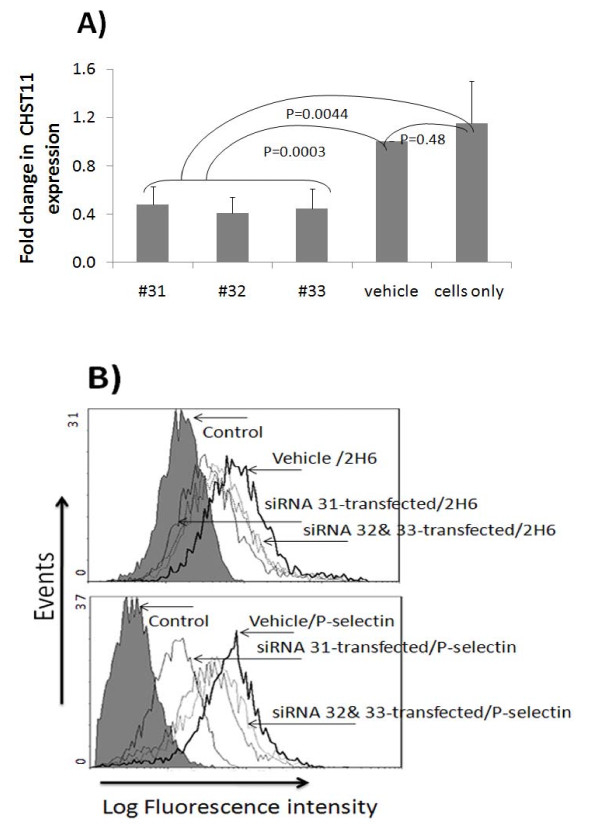
**Inhibition of CHST11 expression and P-selectin binding to MDA-MB-231 cells by *CHST11 *siRNA**. MDA-MB-231 cells were treated with three different siRNAs for the *CHST11 *gene. RNA was harvested after 48 hours and gene expression was assayed at the mRNA level **(A)**. GAPDH was used as the house keeping gene to normalize mRNA-based expression data using the delta delta CT method. CHST11 mRNA levels are shown relative to mRNA level in cells treated with transfection agent only (vehicle). Data were log transformed and subjected to one way ANOVA with post-hocTukey's analysis. **B) **Binding of anti-CS-A (2H6 mAb) (top) and P-selectin (bottom) was tested at Day 6 post siRNA transfection. Binding of secondary antibodies only serves as control. Binding of anti-CS-A 2H6 mAb and recombinant human P-selectin to vehicle-treated and siRNA-treated MDA-MB-231 cells with the three siRNAs is shown. Mean fluorescent intensities of three independent experiments were log transformed and analyzed by ANOVA and post-hoc comparison. Treatment with CHST11 siRNA #31 significantly reduced mean fluorescent intensities for anti-CS-A 2H6 mAb (*P *≤ 0.015) and P-selectin (*P *≤ 0.001) binding, as compared with vehicle-treated cells.

Our data indicate that *CHST11 *is required for P-selectin binding. To confirm a role of 4-O sulfated structures in P-selectin binding, the expression of *CHST11 *in MDA-MB-231 cells was inhibited by siRNA. We observed that *CHST11 *mRNA levels, anti-CS-A binding and P-selectin binding were all significantly reduced upon treatment with the three siRNAs tested (Figures 3A, B). Transfection with siRNA # 31 (Table 1) showed the highest inhibitory effect on binding of anti-CS-A and recombinant P-selectin (Figure 3B). In three independently conducted experiments, transfection with siRNA #31 significantly reduced the mean fluorescence intensity for anti-CS-A (*P *≤ 0.015) and P-selectin (*P *≤ 0.001) binding, as compared with vehicle-treated cells.

We repeated the CHST11 siRNA assay and did not observe any effect on the *CSPG4 *transcript or its surface expression as assayed by anti-CSPG4 mAb (Additional file 1). GAPDH siRNA was used as control in siRNA assays and reduced *GAPDH *mRNA by 75% in multiple assays (data not shown). Treatment of MDA-MB-231 cells with *GAPDH *siRNA did not inhibit *CHST11 *expression (data not shown). *CHST11 *siRNA sequences did not affect expression of *GAPDH *(data not shown). Therefore the expression of CS-A and binding of P-selectin to this cell line depends on the expression of the *CHST11 *gene.

### CSPG4 expressed on aggressive breast cancer cells functions as a P-selectin ligand through its CS-GAGs

**Figure 4:**
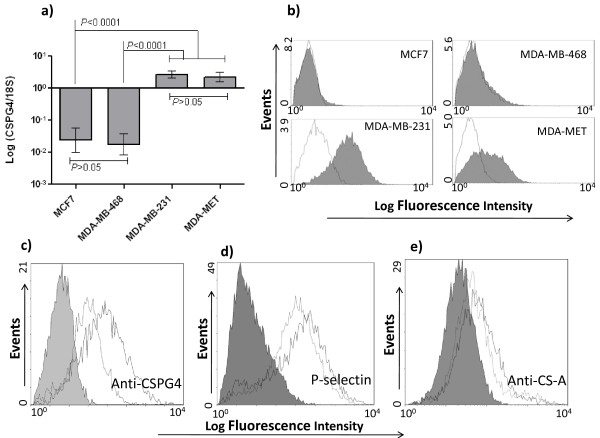
**The expression of CSPG4 in aggressive breast cancer cell lines contributes to P-selectin binding**. **A) ***CSPG4 *mRNA was measured by qRT-PCR and normalized to 18S values and log transformed. Means and standard deviations are shown. Comparisons were made by ANOVA and post-hoc analysis. **B) **Cell surface expression of CSPG4 was examined in the indicated breast cancer cell lines by flow cytometry using anti-CSPG4 225.28 mAb (10 μg/ml). Open histogram shows binding of the secondary antibody only, while filled histogram shows anti-CSPG4 225.28 mAb binding. One representative experiment out of three is shown. **C) **Expression of CSPG4 was inhibited by transient transfection of MDA-MB-231 cells with CSPG4 siRNA that led to a decrease in the binding of P-selectin **(D) **and anti-CS-A **(E) **to transfected cells In C, D and E the filled histograms show the binding of secondary antibodies only (control), the open histograms with solid lines show binding to vehicle-treated cells while the open histograms with dotted lines show binding to the siRNA-transfected cells.

Because CS/DS-GAG expression and function depends on the composition of PGs expressed, studying the nature of the PG(s) involved in presentation of P-selectin-reactive CS/DS is important. Such studies should help us understand the functional consequences of CS/DS-GAG, PG and P-selectin interactions and provide data that may, in future studies, be used to manipulate the expression of the polysaccharide by targeting the core protein(s). Several membrane PGs, including SDC-1, SDC-4, NRP-1, and CSPG4 can potentially present GAG chains on the surface of tumor cells [53-55]. CSPG4 is the only cell surface PG that is exclusively decorated with CS-GAGs [41] and therefore, it may play a major role in forming cell surface CS-GAGs. We compared the expression of *CSPG4 *in the above described human breast cancer cell lines. The results of qRT-PCR indicate that the less aggressive epithelial-like cell lines MCF7 and MDA-MB-468 did not express *CSPG4*, while the gene was highly expressed in the highly aggressive mesenchymal-like cell lines MDA-MB-231 and MDA-MET (Figure 4A). Flow cytometry analysis further confirmed that the expression of CSPG4 was high in aggressive cell lines MDA-MB-231 and MDA-MET with almost no expression detected in the less aggressive cell lines MCF7 and MDA-MB-468 (Figure 4B).

Because CSPG4 is abundant on aggressive cells, we hypothesized that it may function as a major core protein presenting CS-A and CS-related P-selectin ligands. We used *CSPG4 *siRNA to inhibit *CSPG4 *expression (Additional file 2). Inhibiting *CSPG4 *transcript in turn inhibited cell surface expression of the PG (Figure 4C), and that led to a drop in P-selectin (Figure 4D) and anti-CS-A binding (Figure 4E). We examined the transcript levels for *CHST11 *after transfection with *CSPG4 *siRNA (Additional file 2). Inhibition of expression of *CSPG4 *did not affect the expression of *CHST11*, indicating independence of expression of these two genes. This suggests that a decrease in CS-A after *CSPG4 *siRNA treatment (Figure 4E) is due to reduced expression of *CSPG4 *and not of *CHST11*. These data suggest that CSPG4 participates in forming P-selectin ligands on the surface of highly aggressive human breast cancer cells, but obviously it is not the only PG involved in CS-GAG presentation. Others have reported the expression of syndecans and NRP-1 in these breast cancer cell lines and it is known that these PGs can present CS-GAGs [36-38,54-56]. To confirm, we further examined the expression of SDC-1, SDC-4, NRP-1, and CSPG4 by qRT-PCR (Table 3). The expression of SDC-1 was lower in cells with highest metastatic potential and the expression of NRP-1, SDC-4 and CSPG4 was significantly higher in the most aggressive cells. The abundance of CS-A expression in MDA-MB-468 (a cell line with intermediate aggressiveness (Figure 2A), combined with the lack of CSPG4 expression in this cell line (Figure 4A, B), suggest that CS chains on this cell line are probably presented by other PGs (Table 3).

**Table 3:** Ratio of mRNA for some PG genes to 18S RNA

Antigen	MCF7	MDA-MB-468	MDA-MB-231	MDA-MET
*CSPG4*	0.02 (± 0.006)	0.03 (± 0.02)	**2.43 (± 0.56)**	**2.15 (± 0.8)**
*NRP-1*	0.96 (± 0.10)	1.00 (± 0.08)	**1.55 (± 0.20)**	**1.96 (± 0.07)**
SDC-1^*^	0.45 (± 0.07)	**2.09 (± 0.18)**	**0.22 (± 0.03)**	**0.13 (± 0.04)**
SDC-4^**^	0.58 (± 0.08)	**0.86 (± 0.07)**	**1.50 (± 0.30)**	**1.90 (± 0.11)**
ER1	**4.84 (± 0.59)**	0.12 (± 0.03)	0.14 (± 0.03)	0.11 (± 0.02)

Gene expression was measured by qRT-PCR assays, repeated 3 times for each gene and each cell line and averages (± standard deviations) are shown. One-way ANOVA with Tukey's post-hoc procedure on log-transformed data was used for statistical analysis as described in Materials and Methods. Bold entries are statistically different (at *P *< 0.05) than non-bold entries in each row. *The expression of SDC-1 in MDA-MB-468 cells is significantly higher than that in MDA-MB-231 and MDA-MET cells. **The expression of SDC-4 in MDA-MB-468 cells is significantly less than that in MDA-MB-231 and MDA-MET cells. The expression of ER1 was used as a control knowing that MCF7 is ER-positive and the other three cell lines are ER-negative. CSPG4, Chondroitin Sulfate Proteoglycan 4; ER1, Estrogen Receptor 1; NPR-1: Neuropilin-1; SDC-1, Syndecan-1; SDC-4, Syndecan-4.

**Figure 5:**
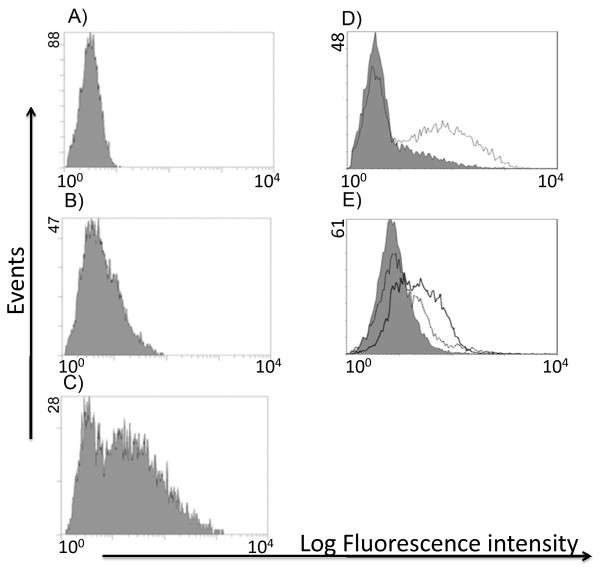
**Expression of CSPG4 leads to an increase in CS-A expression and P-selectin binding**. **A) **Secondary Ab binding (control) to CSPG-4-transfected M14 (M14-CSPG4). Anti-CSPG4 (mAb 225.28) binding to M14-mock-transfected is minimal **(B)**, while the binding is high to CSPG4-transfected cells **(C)**. **D) **Overlay histogram of 2H6 mAb (anti-CS-A) binding to the M14-mock-transfected (filled histogram) and M14-CSPG4 cell line (open histogram). **E) **P-selectin binds to M14-CSPG4 (open histogram, solid line) and not to M14-mock-transfected (filled histogram). Binding of P-selectin to M14-CSPG4 is reduced after treatment with chondroitinase ABC (dotted line shifted to the left). The experiment was repeated three times and one representative is shown.

In order to confirm that P-selectin binds to CSPG4 we used CSPG4-transfected M14 melanoma cell line available in the lab. In prescreening of the M14 cell line, it appeared that this cell line expresses *CHST11 *with low binding of anti-CS-A 2H6 mAb and P-selectin, indicating that this cell line and its transfected version are excellent candidates for studying participation of CSPG4 and its CS GAGs in P-selectin binding. We observed that anti-CSPG4 225.28 mAb reacted with the transfected cells (M14-CSPG4) but not with mock-transfected M14 cells (Figure 5A, B, C). Anti-CS-A 2H6 mAb reacted with M14-CSPG4 but not with mock-transfected M14 cells (Figure 5D). P-selectin also reacted with M14-CSPG4 cells but not mock-transfected M14 cells, and treatment of M14-CSPG4 cells with chondroitinase ABC reduced the reactivity (Figure 5E). Pre-incubation of cells with 225.28 did not inhibit P-selectin binding (data not shown). These data suggest that CSPG4, via its CS chain, serves as a P-selectin ligand on the cell surface and that anti-CSPG4 225.28 mAb does not react with the GAG part of CSPG4. The data further suggest that overexpression of both *CSPG4 *and *CHST11 *genes in tumor cells may contribute to a superior metastatic potential.

### *CHST11 *and *CSPG4 *are overexpressed in malignant tissues of breast cancer patients

**Figure 6:**
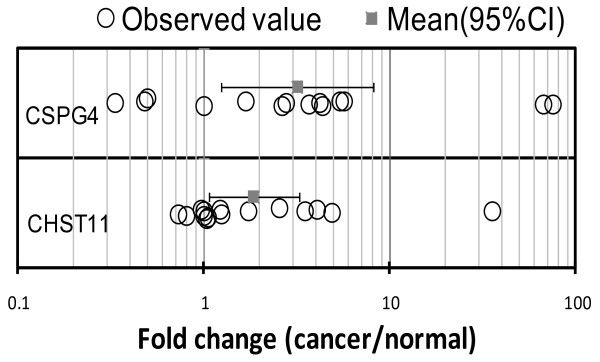
**Expression of the *CSPG4 *and the *CHST11 *genes was detected in breast tissue samples**. mRNA expression was quantified by absolute quantification and the ratio of mRNA to 18S mRNA was calculated. The fold change in tumor sample compared to normal tissue sample in each subject was calculated and plotted. Circles denote individual observations, while squares with error bars represent group means with their 95% confidence intervals (CIs). *CSPG4 *and *CHST11 *were elevated 3.2 (*P *< 0.02) and 1.8 (*P *= 0.034) fold, respectively in tumor-containing samples over normal samples.

In order to establish a translational relevance, we examined the expression of *CSPG4 *and *CHST11 *in specimens from breast cancer patients to compare the level of expression of these genes between normal and malignant tissues. Frozen sample-pair specimens from 15 breast cancer patients diagnosed with invasive ductal carcinoma were obtained from the UAMS tissue bank. In each sample pair, a tumor-containing sample was matched with tumor-free tissue from the same donor. We observed that these genes were overexpressed in tumor-containing tissues versus normal tissues (Figure 6). *CSPG4 *and *CHST11 *expression showed an increase in tumor tissue over normal tissue in 10 out of 14 sample pairs and 8 out of 15 sample pairs, respectively. *CSPG4 *was elevated 3.2-fold (*P *< 0.02) in tumor tissue over normal tissue among 14 subjects, while *CHST11 *was elevated 1.8-fold (*P *= 0.034) in tumor over normal among 15 subjects (Figure 6). Gene overexpression was detected in both ER-positive and ER-negative samples, but the majority of specimens with increased expression of *CHST11 *(seven out of eight), were HER2-neu-negative. These data suggest that despite the stromal expression of both *CHST11 *and *CSPG4 *genes the expression can be targeted specifically in aggressive tumors for therapeutic purposes. In this regard P-selectin binding to a relevant receptor on tumor cells may have profound impact on tumor cell dissemination and understanding the detail of such interaction may lead to development of novel anti-metastatic approaches.

### CS-GAG removal inhibits tumor metastasis

**Figure 7:**
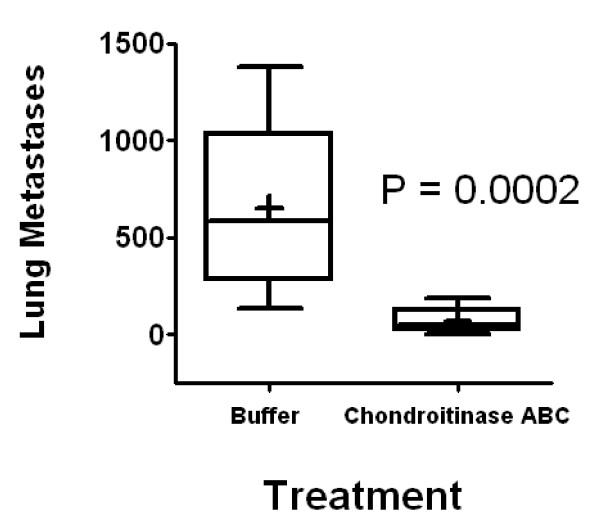
**Enzymatic removal of surface CS-GAGs reduced lung colonization of 4T1 cells**. 4T1 cells were treated with chondroitinase ABC or buffer and injected into the tail vein of BALB/c mice (10 per group). Mice were sacrificed 25 days later and the number of metastases to the lung was measured by clonogenic assay and expressed as "Lung Metastases". Boxes show medians and quartiles while whiskers show ranges; plus signs indicate means. *P *= 0.0002 by Wilcoxon rank-sum test.

We have previously suggested a role for CS/DS-GAGs in metastasis of the murine mammary cell line 4T1 [24]. To directly link CS/DS-GAGs to tumor metastasis, we examined whether removal of the cell surface CS/DS-GAGs affects metastasis of 4T1 cells *in vivo*. Here we demonstrate that removing CS/DS-GAGs by treating cells with chondroitinase ABC attenuated lung metastases in the 4T1 murine tumor model (Figure 7). Chondroitinase treatment of cells prior to tail-vein injection significantly reduced lung metastases (*P *= 0.0002). The data indicate a significant role for tumor surface CS/DS-GAGs in establishing lung metastases in this breast cancer model and further support a likely role for P-selectin interaction with CS/DS-GAG in breast cancer metastasis.

## Discussion

Tumor cell dissemination by platelets leads to colonization of cancer cells to secondary organs resulting in poor prognosis and high mortality of cancer patients. P-selectin is present on activated platelets and endothelial cells while CS/DS-GAGs on the surface of breast cancer cells with high metastatic potential serve as P-selectin ligands [24]. The role of P-selectin in heterotypic adhesion is a critical component determining the efficiency of tumor cell dissemination [16,17]. The study of P-selectin-reactive molecules on tumor cells is crucial for the assessment of metastatic risk and the development of possible ways of dealing with metastatic disease. Such studies are needed to ultimately reveal the functional consequences of P-selectin/ligand interaction in tumor progression, making specific links between platelets and tumor metastasis.

Our results suggest the *CHST11 *gene as a major player in production of such P-selectin ligands. The results using *CHST11 *siRNA further suggest that among the chondroitin sulfotransferases tested, *CHST11 *expression has a rate limiting role in constructing both CS-A chains and P-selectin ligands on MDA-MB-231 cells. This data directly links expression of the *CHST11 *gene to P-selectin-reactive GAGs on this cell line and has significant implications for further functional studies. CS-A is an immediate product of *CHST11 *expression and is considered a precursor in forming CS-E units [57]. P-selectin has been shown to bind to CS-E [23]. We have also shown that exogenous CS-E inhibits P-selectin binding to cancer cells [24]. While we do not rule out participation of CS-E in P-selectin binding, our data do not support CS-E as the P-selectin reactive GAG unit on these cells. The combined high expression of these two genes may associate with P-selectin-reactive glycans and aggressiveness. This possibility should be investigated in future studies. However, the data indicate that the expression of the *CHST11 *gene in tumor cells is associated with synthesis of P-selectin ligands and a metastatic phenotype. Others have suggested a role for CS-A in tumor progression and metastasis in a melanoma model [46]. However, our data suggest that surface presentation of CS-A may be required but is not sufficient for a metastatic phenotype to occur. Besides its role in constructing CS-A, CHST11 also plays a role in chain elongation and production of more CS [57]. Chain elongation activity of CHST11 with a fine balance in the expression of other enzymes might be needed for constructing conformational epitopes. Thus, CHST11 activity may lead to larger CS polymers with multiple sequences embedded with distinct sulfation patterns resulted from activity of multiple sulfotransferases, leading to the production of conformational epitopes or highly concentrated sequences with specific reactivities. Thereby, the expression of *CHST11 *may correlate better with the tumor cells' aggressive phenotype than does the prevalence of any particular CS isomers.

Interestingly, our current results implicate CSPG4 in the presentation of CS moieties as P-selectin ligands. CSPG4 exclusively presents CS-GAGs, and the data described here suggest that these structures interact with P-selectin and, therefore, may contribute to distant metastasis of tumor cells. Our data indicate that P-selectin binds to CSPG4 through CS-GAGs and that CSPG4 is involved in P-selectin binding to CSPG4-expressing breast cancer cells. However, other PGs may also participate in P-selectin binding as expression of SDC-4 and NRP-1 is also higher in MDA-MB-231 and MDA-MET. Lack of expression of CSPG4 in MDA-MB-468 further suggests that anti-CS-A and P-selectin binding to these cells is probably due to expression of other PGs and not CSPG4. However, because of the role of CSPG4 in signaling and tumor phenotype, we speculate that its interaction with P-selectin may lead to an exclusive tumor cell activation and consequently survival in circulation. Therefore, concerted upregulation of *CSPG4 *and *CHST11 *may induce expression of a unique molecular entity that may increase the metastatic capabilities of tumor cells. More studies are needed to understand the consequences of P-selectin binding to CS-GAGs of multiple PGs and to reveal how and at what stage of the metastatic cascade the CS-GAGs and their carrier proteins contribute to metastasis.

We have shown glycan interactions with P-selectin and the significance of P-selectin binding in metastasis of a murine mammary cell line [24,52]. Our previous findings support the concept that CS chains promote survival in the circulation and tumor cell extravasation via P-selectin-mediated binding to platelets and endothelial cells. In the current study, the significance of the cell surface expression of CS-GAGs in a breast cancer model is established. The data demonstrate that enzymatic removal of the CS chains significantly attenuated formation of lung metastases in a highly metastatic mammary cell line. Others have shown that P-selectin ligands are critical components of heterotypic adhesion, determining the efficiency of tumor cell dissemination [16,17]. Sugahara's group demonstrated that highly sulfated CS-GAGs, in particular CS-E, are involved in metastasis of murine lung carcinoma and osteosarcoma cells [58,59]. However, our data suggest a role for 4-O sulfation of chondroitin in the metastatic phenotype. Moreover, the data suggest that the presence of CS/DS-GAGs may not be sufficient for a phenotype with high metastatic capacity to occur. The data emphasize a combination of the polysaccharide and a core protein as a pro-metastatic entity. Future studies are needed to understand the contribution of each PG in P-selectin mediated tumor cell behavior.

We further showed that the expression of *CHST11 *and *CSPG4 *is elevated in tumor tissues from breast cancer patients. Consistent with our data, *CHST11 *expression has been shown to be greater in human breast carcinoma compared to normal breast tissue [60] and in malignant plasma cells from myeloma patients compared to normal bone-marrow plasma cells [61].

The current research should lead to future studies of functional relationships between CS and tumor progression. Existing knowledge and further mechanistic studies might suggest CS-GAGs and their presenting PGs as targets for antimetastatic therapies. In support of work done in melanoma [62] and recent studies in breast cancer [39], the studies outlined here strongly suggest that CSPG4 can be an available target for immunotherapy of breast cancer. However, in order to efficiently block tumor cell dissemination by interrupting P-selectin/CS interaction, targeting any single PG does not seem enough as other PGs can probably compensate and support metastatic processes. In this regard, global targeting of specific CS isomers may be a particularly effective approach.

Breast cancer cell surface is decorated with CS-GAGs and due to tumor-specific expression patterns of chondroitin sulfotransferases and PGs, the composition and binding specificity of these polysaccharides differ from those of normal tissues. Therefore, these molecules and their interaction with P-selectin should be considered as viable targets for the development of novel therapeutic strategies.

## Conclusions

This study demonstrates the significance of CS-GAGs in the lung colonization of an aggressive murine mammary cell line. The study reveals that CSPG4 can serve as a P-selectin ligand through its CS chain and that the expression of the *CHST11 *gene controls P-selectin reactive CS-GAGs formation. The data suggest that CS-GAGs, their biosynthetic pathway, or the core protein carrying them can be potential-targets for the development of therapeutic strategies for treatment of aggressive breast tumors. The knowledge and perspective gained from this line of research together with further mechanistic studies may pave the road to target CS-GAGs, their carrier PGs and their interaction with P-selectin as novel antimetastatic therapies.

## Abbreviations

18S: 18S ribosomal RNA; ANOVA: Analysis of Variance; CS: Chondroitin Sulfate; CS-GAGs: Chondroitin Sulfate Glycosaminoglycans; CS/DS: Chondroitin Sulfate/Dermatan Sulfate; CS/DS GAGs: Chondroitin Sulfate/Dermatan Sulfate Glycosaminoglycans; CHST3: Carbohydrate (chondroitin 6) sulfotransferase 3; CHST7: Carbohydrate (N-acetylglucosamine 6-O) sulfotransferase 7; CHST11: Carbohydrate (Chondroitin 4) Sulfotransferase 11; CHST12: Carbohydrate (Chondroitin 4) Sulfotransferase 12; CHST13; Carbohydrate (Chondroitin 4) Sulfotransferase 13; CHST14: Carbohydrate (N-acetylgalactosamine 4-O) Sulfotransferase 14; CHST15: Carbohydrate (N-acetylgalactosamine 4-sulfate 6-O) Sulfotransferase 15; CS-A: Chondroitin Sulfate A unit; CS-E: Chondroitin Sulfate E unit; CSPG4: Chondroitin Sulfate Proteoglycan 4; DS: Dermatan Sulfate; DS4S-1: Dermatan 4-sulfotransferase 1; ER1: Estrogen Receptor 1; GAGs: Glycosaminoglycans; GalNAc: N-acetyl-D-galactosamine; GalNAc4S-6ST: N-acetylgalactosamine 4-sulfate 6-O-sulfotransferase; GAPDH: Glyceraldehyde-3-phosphate dehydrogenase; GlcNAc: N-acetyl-D-glucosamine; GlcA: Glucuronic acid; IdoA: Iduronic acid; mAb: monoclonal Antibody; NPR-1: Neuropilin-1; PG: Proteoglycan; qRT-PCR: Quantitative Real-Time Polymerase Chain Reaction; SDC-1: Syndecan-1; SDC-4: Syndecan-4; siRNA: short interfering RNA; UAMS: University of Arkansas for Medical Sciences.

## Competing interests

BMK and TKE are named as inventors on an institutional patent application filled by UAMS that is related to the content of this manuscript. No financial or other support of any kind has resulted from this patent application. The other authors declare that they have no competing interests.

## Authors' contributions

CAC participated in data interpretation, and was involved in drafting, critically reviewing and revising the manuscript. FJ carried out tissue culture, animal experiments, cell treatment and flow cytometry assays. AYB participated in the design of real-time PCR assays and helped draft the manuscript. BP carried out siRNA and real-time PCR assays. TG and AMKE carried out the additional gene expression analyses added to the revised manuscript. ERS performed statistical analyses and helped draft the manuscript. SF, TKE, and LJS participated in design of the study and helped draft the manuscript. BMK conceived of the study, designed experiments, coordinated the study and drafted the manuscript. All authors deserve the authorship right and they read and approved the final manuscript.

## Supplementary Material

Additional file 1 **Supplemental Figure S1**. Transient transfection of MDA-MB-231 cells with *CHST11 *siRNA inhibits *CHST11 *expression **(A)**, anti-CS-A **(C) **and P-selectin **(D) **binding with no effect on CSPG4 mRNA **(B) **or the surface expression of the PG **(E)**. Click here for file

Additional file 2 **Supplemental Figure S2**. Fold change in the expression of *CHST11 *and *CSPG4 *mRNA after transient transfection with *CSPG4 *siRNA. The expression of genes was measured 48 hours post transfection by qRT-PCR. Fold change is calculated based on the expression of genes in vehicle-treated cells. GAPDH message was used to normalize the data. Transfection with a scrambled siRNA was used as additional control. **, significantly different than expression in either cells only or cells transfected with scrambled siRNA at *P *< 0.01. Click here for file
