# Supplementary figures and images for: Chondroitin sulfates play a major role in breast cancer metastasis: a role for CSPG4 and CHST11 gene expression in forming surface P-selectin ligands in aggressive breast cancer cells
Source: Breast Cancer Res. 2011 Jun 9;13(3):R58. doi: 10.1186/bcr2895 (PMC3218947; doi:10.1186/bcr2895)

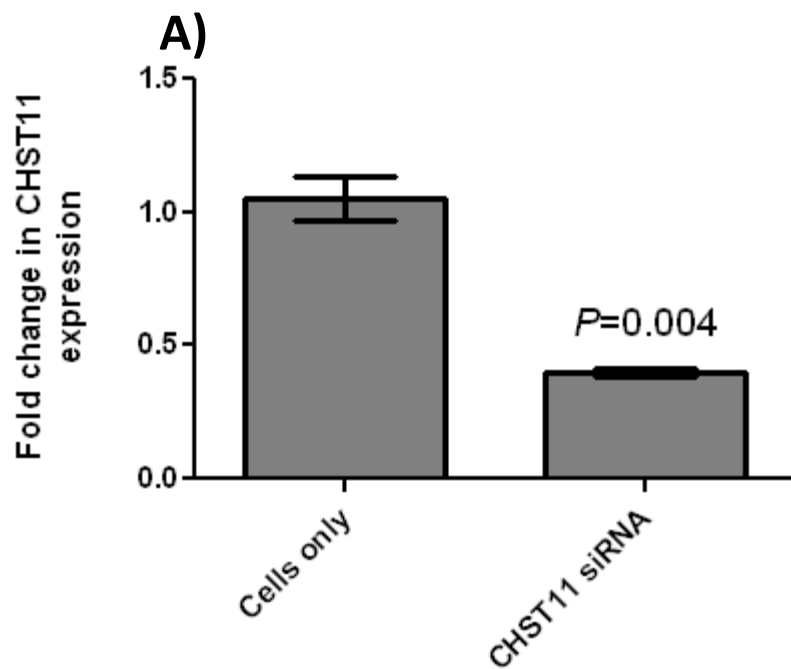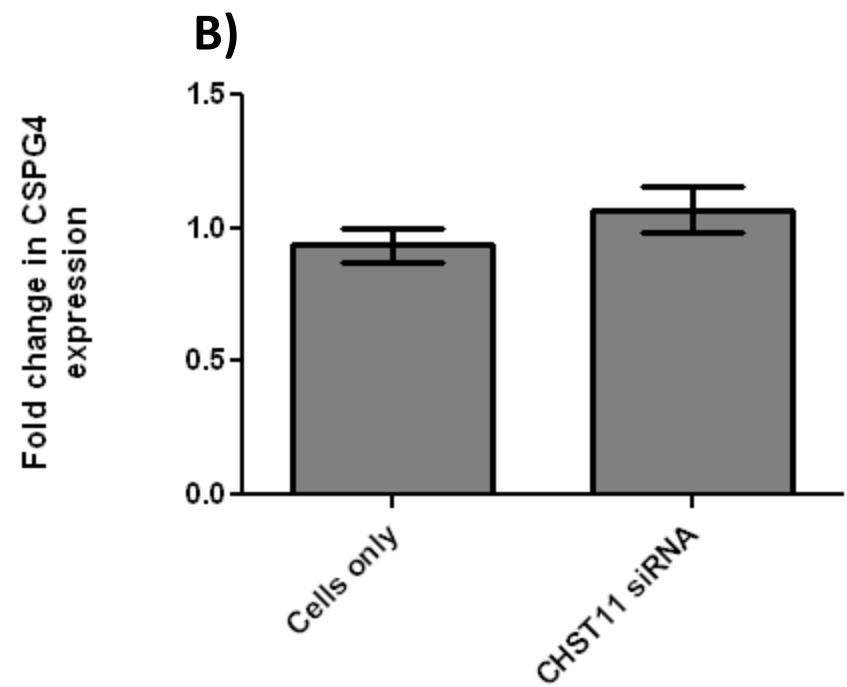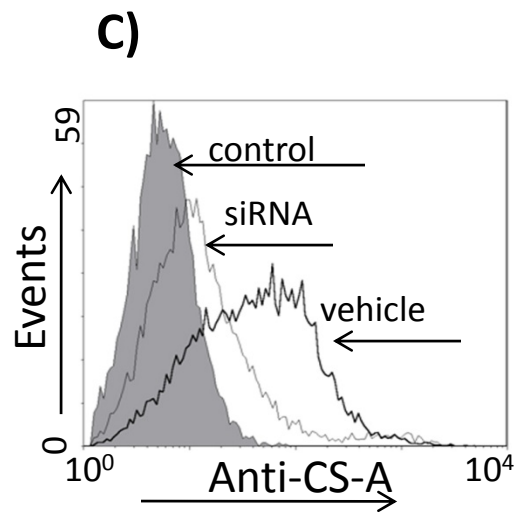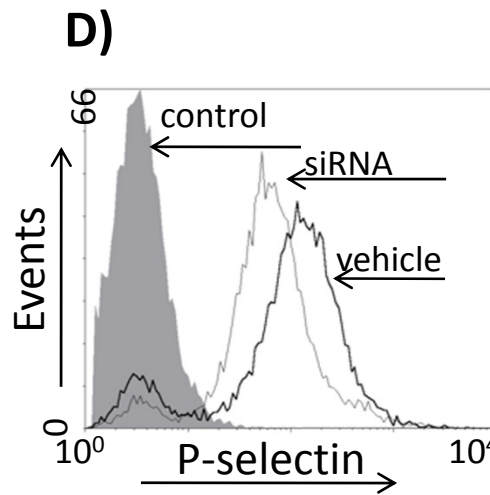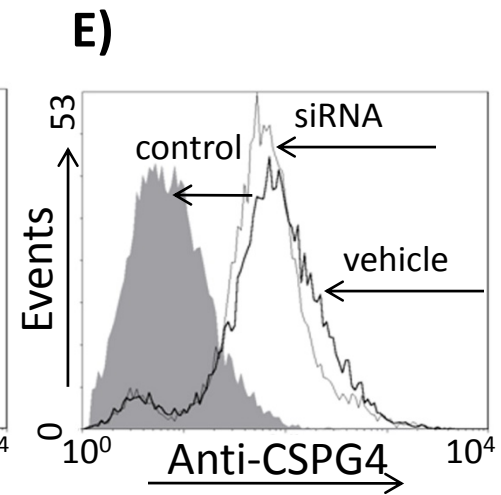

**Figure 1S**

Supplement: Additional file 1 — Supplemental Figure S1. Transient transfection of MDA-MB-231 cells with CHST11 siRNA inhibits CHST11 expression (A), anti-CS-A (C) and P-selectin (D) binding with no effect on CSPG4 mRNA (B) or the surface expression of the PG (E). [file bcr2895-S1.PDF]

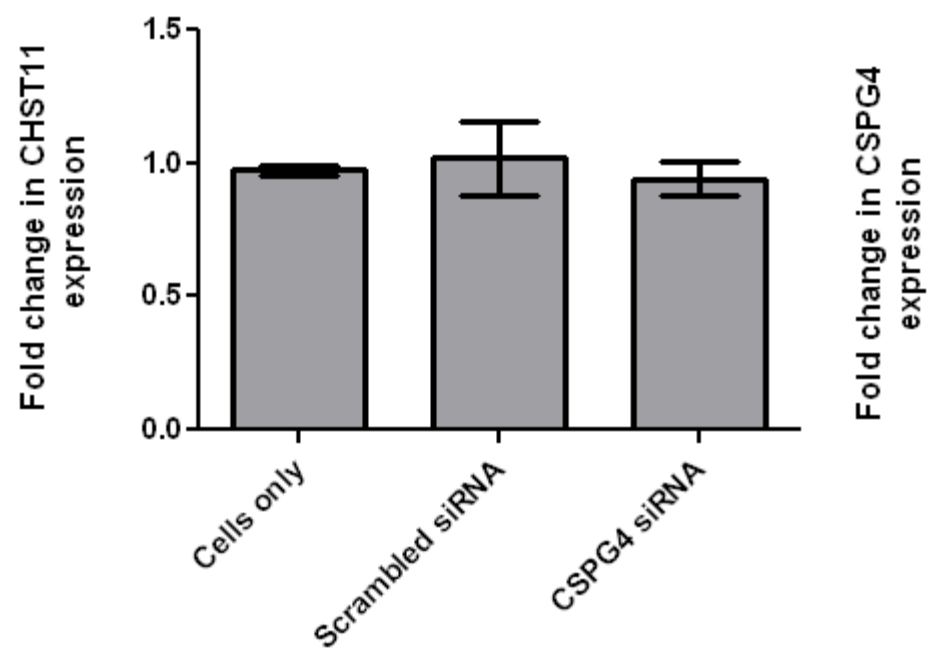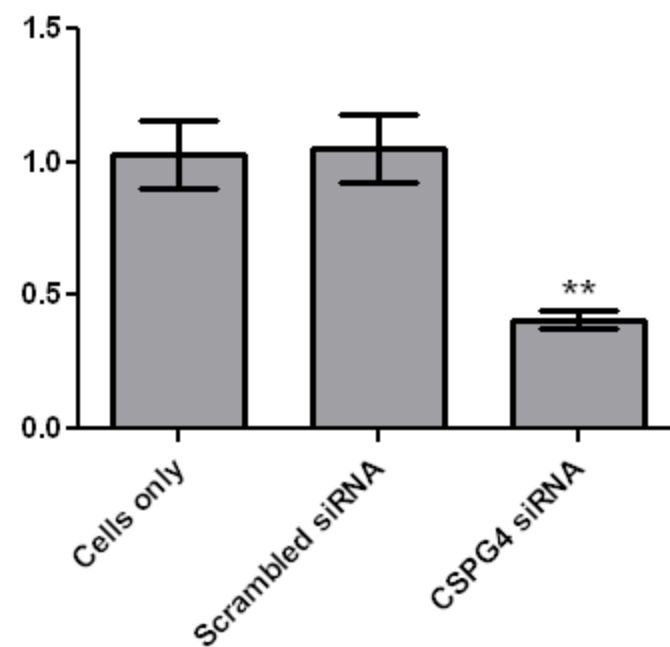

Figure 2S

Supplement: Additional file 2 — Supplemental Figure S2. Fold change in the expression of CHST11 and CSPG4 mRNA after transient transfection with CSPG4 siRNA. The expression of genes was measured 48 hours post transfection by qRT-PCR. Fold change is calculated based on the expression of genes in vehicle-treated cells. GAPDH message was used to normalize the data. Transfection with a scrambled siRNA was used as additional control. **, significantly different than expression in either cells only or cells transfected with scrambled siRNA at P < 0.01. [file bcr2895-S2.PDF]
